# Ramsay Hunt Syndrome With Multiple Cranial Neuropathies, Meningitis, and Subsequent Brainstem Encephalitis: A Case Report

**DOI:** 10.7759/cureus.73861

**Published:** 2024-11-17

**Authors:** Satoshi Kawamoto, Kazuhiro Yoshinaga, Ryosuke Watanabe, Takashi Hirano

**Affiliations:** 1 Otolaryngology, Oita University, Yufu, JPN; 2 Neurology, Oita University, Yufu, JPN

**Keywords:** brainstem encephalitis, cranial neuropathies, meningitis, ramsay hunt syndrome, varicella-zoster virus

## Abstract

We present a case of Ramsay Hunt syndrome in a previously healthy 49-year-old male, complicated by ipsilateral glossopharyngeal, vagus, accessory, and hypoglossal nerve palsies, along with meningitis. Despite a course of antiviral therapy and steroids for meningitis, the patient experienced a relapse, developing varicella-zoster virus (VZV) brainstem encephalitis after an initial period of stability. Hunt syndrome can be encountered in otolaryngology and internal medicine, dermatology, and other specialties. We report a case of Ramsay Hunt syndrome complicated by multiple cranial neuropathies and Hunt syndrome-associated meningitis and encephalitis, accompanied by a literature review.

## Introduction

Ramsay Hunt syndrome (Hunt syndrome) is characterized by herpes zoster oticus, facial nerve paralysis, and eighth cranial nerve dysfunction due to the varicella-zoster virus's (VZV) reactivation. J. Ramsay Hunt first described this syndrome in 1907 [1]. The causative virus of Hunt syndrome, VZV, can lead to various cranial neuropathies, particularly in immunocompromised patients. Although rare, cases of multiple cranial neuropathies have been reported in Hunt syndrome [2]. VZV can cause central nervous system infections, such as meningitis, especially in immunocompromised individuals [3]. 

## Case presentation

**Figure 1:**
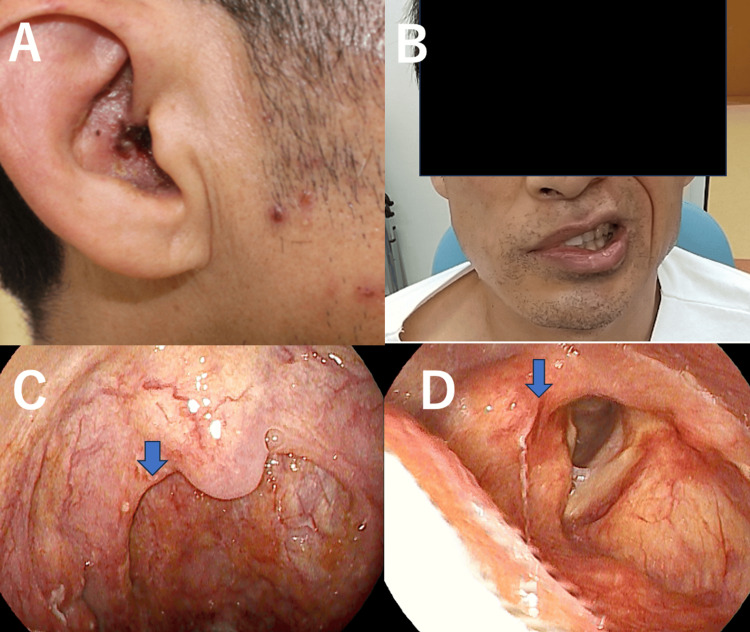
Rash was observed on the right side of the face (A). Complete paralysis of the right side of the face was observed (B). The right curtain sign was positive, and the right vocal cord had reduced mobility (C, D).

The patient is a 49-year-old male with no notable medical, personal, or family history. Five days prior to his first visit to our department, he felt pain in his right ear and was diagnosed with otitis externa by an otolaryngologist. Then, dizziness and a rash on the right auricle appeared, and he returned to the clinic, which suspected Hunt's syndrome and was prescribed valacyclovir, 3000 mg per day. The following day, facial paralysis appeared, and he was subsequently referred to our department. He was alert on physical examination, with a body temperature of 37.6°C. A rash was observed on the right facial area, right pinna, and the right external auditory canal (Figure 1A). Horizontal rotatory nystagmus to the left was noted. Hypoesthesia was present in the right facial and pinna areas. Right facial paralysis was evident, scoring 0 on the Yanagihara Facial Nerve Paralysis Scale [4]. Grade VI on the House-Brackmann scale (Figure 1B) [5]. A right-sided oral mucosal rash was observed, and the right curtain sign was positive. There was reduced mobility of the right vocal cord (Figures 1C, 1D).

**Figure 2:**
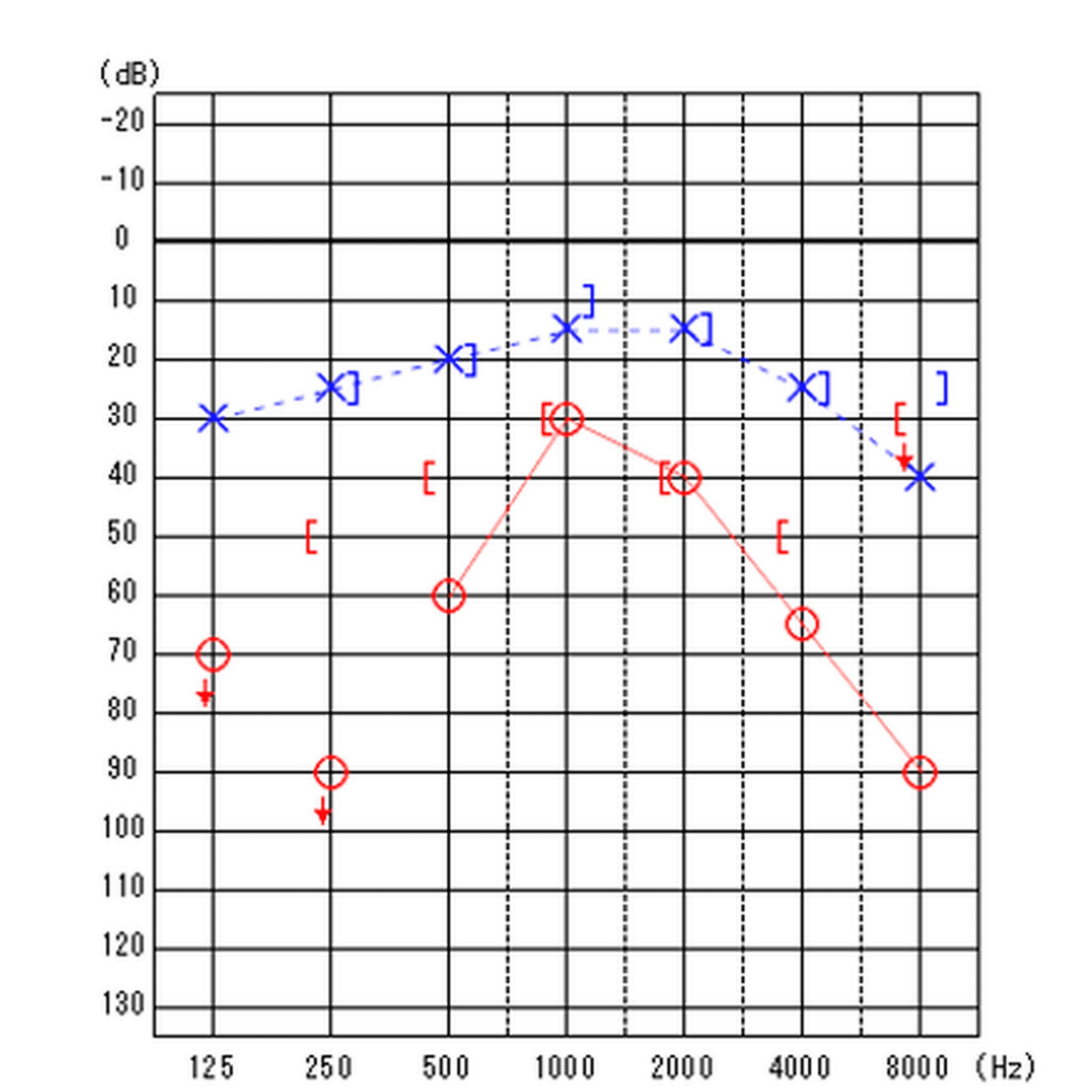
Right-sided sensorineural hearing loss is observed.

The right side of the tongue exhibited poor movement, and the right sternocleidomastoid muscle strength was decreased. Slight neck stiffness was also noted. He was unable to walk and required a wheelchair for mobility. Standard pure tone audiometry revealed moderate sensorineural hearing loss on the right side at 40.0 dB and on the left at 14.4 dB (Figure 2).

**Figure 3:**
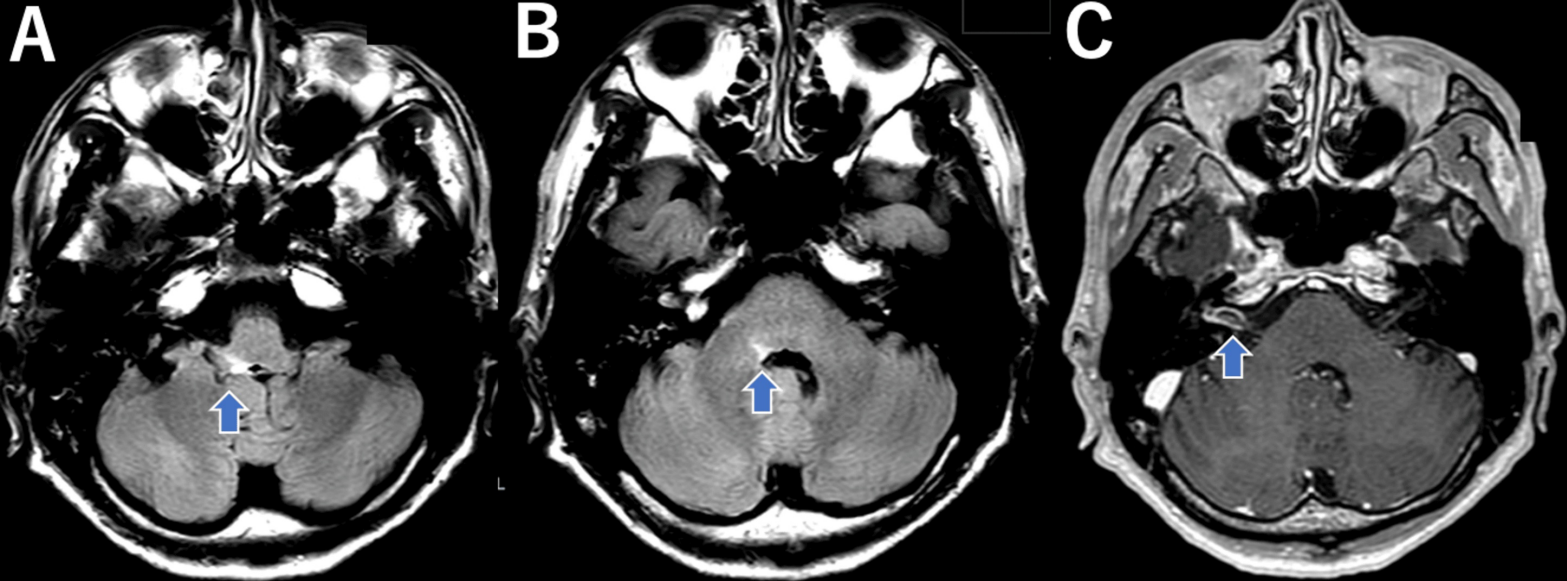
Head MRI, FLAIR sequence, showed high signal intensity in the right vestibular nuclei and surrounding areas. On contrast-enhanced MRI, enlargement of the facial nerve was observed.

The stapedius reflex was unresponsive on the right side. The electric taste test showed scale-out results in the chorda tympani, glossopharyngeal regions, and greater petrosal nerves, exceeding 34 dB. On contrast-enhanced MRI of the head, no abnormalities were observed in the internal auditory canal. No other notable findings were observed. Blood tests and cerebrospinal fluid findings are presented in separate tables. He was diagnosed with aseptic meningitis and herpes zoster oticus and was administered antiviral therapy (acyclovir 30 mg/kg/day) and hydrocortisone 750 mg/day intravenously from the first day of hospitalization. On day 3 of treatment, the accessory nerve palsy, hypoglossal nerve palsy, and vocal cord palsy showed improvement. Oral intake became possible; on day 4, facial nerve palsy improved to 8 points and pain around the right auricle also improved; on day 8, subjective symptoms of dizziness almost disappeared; on day 14, spinal fluid examination showed normalization of cell count, and treatment of meningitis was terminated with discontinuation of acyclovir; On day 16, the patient complained of right occipital pain, and pregabalin was started on suspicion of neuropathic pain. Although further exacerbation of dizziness was observed, the nystagmus findings indicated irritative nystagmus, and the patient was considered to be recovering from paralytic nystagmus caused by Hunt's syndrome. On day 17, the patient was deemed ready for discharge and was scheduled to return home. On the 24th day following the initial admission, the patient was readmitted to our hospital due to worsening fatigue, dizziness, and difficulty in walking and taking oral intake. Physical examination revealed clear consciousness, and body temperature was 36.9°C. A crusted skin rash was observed on the right face and external auditory canal. Left horizontal rotatory nystagmus was observed, and hypesthesia was noted in the right facial area and auricularis. Right facial nerve palsy did not worsen to 6 points. There was reduced muscle tone in the right sternocleidomastoid and trapezius muscles. Bilateral deep tendon reflexes and the Wartenberg reflex were enhanced, suggesting pyramidal signs. Head MRI showed enlargement and contrast enhancement of the right vestibulocochlear nerve, a high signal on FLAIR, and contrast enhancement in the vestibular nucleus. These findings indicate possible brainstem involvement, which could explain the presence of pyramidal signs. Swelling and contrast enhancement were also observed in the right facial and internal auditory nerves (Figure 3). Blood tests and cerebrospinal fluid findings are presented in Table 1.

**Table 1:** Laboratory studies. CRP: C-reactive protein; CSF: cerebrospinal fluid

	First day	Day 14	Day 24 (readmission)	Normal range
White blood cell count (μL)	6290	5070	4930	3100-8400
Neutrophils (%)	82.9	58.4	66.7	41-72
Lymphocytes (%)	12.1	33.7	25.4	21.2-51.0
CRP (mg/dL)	0.13	0.01	0.01	0.00-0.14
CSF cell count (/mm³)	116	8	55	0-5
CSF protein (mg/dL)	80.7	27.5	36.1	8-43
CSF VZV-DNA	15×10⁵ positive	-	Negative	

On the 24th following the initial admission, he was diagnosed with VZV vasculitis and brainstem encephalitis and was urgently admitted to the Department of Neurology. Treatment with antiviral medication (acyclovir 30 mg/kg/day) for 14 days and a five-day course of steroid pulse therapy with hydrocortisone 1000 mg/day were initiated. Additionally, due to the risk of stroke associated with vasculitis, oral administration of aspirin was started. On the 26th day, attempts at vestibular function tests were made, but intense nausea prevented their completion. By the 27th day, he showed improvement and could take oral nourishment. Headaches and symptoms of vertigo showed improvement. By the 30th day, symptoms of vertigo only occurred with body movement, and the patient could walk. By the 36th day, further improvement in symptoms was noted. A repeat MRI of the head showed no change in findings. By the 49th day, all symptoms had improved enough for discharge, except for hearing loss and persistence of vertigo with body movement. The facial nerve score was 12 points at the six-month follow-up, and the House-Brackmann scale was Grade III. Subsequent MRI of the head six months post-discharge showed the disappearance of the high signal area in the vestibular nucleus observed at readmission.

## Discussion

In this case, a healthy adult male with no underlying disease developed meningitis and cranial neuropathy associated with Hunt's syndrome and had to be rehospitalized for brainstem encephalitis. Hunt syndrome is well known to be severe in immunocompromised and elderly patients, but severe cases in immunocompetent adults have rarely been reported [6,7]. Petersen et al. reported an incidence of 1.6 cases per million of Hunt syndrome-associated meningitis in a large cohort study in Denmark [8]. This underscores the rarity of Hunt syndrome complicating meningitis, especially in healthy individuals. This case highlights that even healthy adult males can experience serious complications. Although occasional cases of Hunt syndrome exacerbated by complications of meningitis or encephalitis have been reported, to our knowledge, there have been no reports of cases requiring rehospitalization due to relapse despite adequate duration of treatment [9-11]. This case suggests that the generally recommended duration of treatment may be inadequate to cure and relapse in some cases. In cases of Hunt syndrome with fever and multiple cranial neuropathies, it is essential to recognize the potential involvement of the central nervous system, such as meningitis or encephalitis [12]. If such symptoms are present, it is possible that the disease has progressed beyond the typical Hunt syndrome and is affecting the central nervous system. In such cases, invasion of the CNS should be carefully considered and treated accordingly. This case suggests that patients can develop severe CNS complications even in the absence of obvious risk factors such as immunosuppression, underscoring the need for early detection and aggressive management in similar cases. Although no strict guidelines exist for antiviral therapy for Hunt syndrome, the Infectious Diseases Society of America (IDSA) recommends 14 days for VZV encephalitis [13]. The spinal fluid findings became standard after the treatment, and the patient was cured. The treatment was terminated, but the disease relapsed despite standard 14 days of antiviral therapy. Cerebellar symptoms such as dizziness persisted beyond the 14-day treatment period, suggesting that complete recovery may not have been achieved. This suggests the possibility of reactivation of VZV infection in the central nervous system. Therefore, even after completing a standard course of treatment, it is essential to closely monitor clinical symptoms and evaluate whether additional treatment is warranted. Complications involving other cranial neuropathies, such as trigeminal, glossopharyngeal, and vagus nerves, have been reported in Hunt syndrome, and these cases tend to have a poor prognosis [14,15]. When the patient in this case was first admitted to the hospital, a severe CNS infection, including meningitis, was suspected because of complications involving the trigeminal nerve, glossopharyngeal nerve, vagus nerve, accessory nerves, and hypoglossal nerve. Neuropathy other than the facial and vestibulocochlear nerves improved shortly after treatment, but facial palsy and improved balance function and hearing were not observed. MRI findings on rehospitalization revealed swelling of the entorhinal and facial nerves, suggesting persistent neurological damage due to prolonged inflammation. In the case of facial and vestibulocochlear nerve palsy, microvasculitis affecting nutrient vessels such as the ascending pharyngeal artery is considered a possible mechanism. In this case, VZV vasculitis was suspected, and aspirin was administered acutely to prevent stroke. VZV vasculitis is known to cause granulomatous vasculitis in both large and small cranial vessels, which can lead to stroke. In a retrospective study of patients with VZV meningitis, three of 15 cases reported having a stroke, and in young people and children, one-third of strokes are associated with VZV vasculitis [16,17]. In this case, aspirin was administered to prevent cerebral infarction, and the medication was discontinued after the acute phase. However, the criteria and duration of aspirin administration in severe cases of Hunt syndrome and VZV vasculitis have not yet been established, and further case accumulation and discussion are needed. In Japan, the varicella vaccine became a routine vaccination for children in 2014, and in 2016, the vaccine was approved for people over 50 years of age to prevent herpes zoster [18]. Shingles can cause a variety of sequelae and, as demonstrated in this case, can significantly impair healthy adults' quality of life (QOL). Therefore, prevention through vaccination has become critical. Furthermore, considering the severity of this 49-year-old case, lowering the target age for herpes zoster vaccination needs to be considered.

## Conclusions

We experienced a case where a previously healthy 49-year-old male, initially treated for Hunt syndrome, required readmission due to the development of brainstem encephalitis caused by VZV. This case highlights that even in immunocompetent adults, severe complications such as brainstem encephalitis can occur and relapse despite an adequate initial treatment duration. In cases where central symptoms accompany peripheral facial nerve paralysis, it is essential to differentiate between conditions like meningitis and meningoencephalitis early to ensure timely and appropriate intervention. Moreover, as this case demonstrates, even after completing the recommended course of antiviral and steroid therapy, there remains a risk of recurrence, suggesting that current treatment guidelines may need reassessment or adjustment for cases involving central nervous system complications. Continuous monitoring of clinical symptoms beyond the treatment period is crucial, and consideration should be given to extending therapy or re-evaluating treatment strategies if symptoms persist. Finally, preventive measures, including vaccination against herpes zoster, should be emphasized as they are crucial in minimizing the risk of severe complications and improving the QOL for at-risk adults.
